# Approach for sustainable district-led production and distribution of alcohol-based hand rub in Uganda

**DOI:** 10.1017/ash.2023.224

**Published:** 2023-09-29

**Authors:** Maureen Kesande

## Abstract

**Background:** A sustainable, continuous supply of alcohol-based hand rub (ABHR) is essential for healthcare workers in health facilities. The WHO provides guidance for production in individual health facilities. In Uganda, using this guidance, an innovative approach was implemented at the district local government level to produce and subsequently distribute ABHR to primary-care health facilities that have limited capacity for local facility-level production. This project was supported by the CDC in collaboration with the Infectious Diseases Institute (IDI) and targeted governmental or district engagement with local partners to ensure sustainability. **Methods:** District stakeholders were engaged to obtain buy-in and define roles and responsibilities. Overall, 4 staff members in each of 6 supported districts were nominated by District Health Officers for training: 2 staff members were trained to produce ABHR and conduct internal quality control and 2 were trained on external quality control. Districts provided ABHR production-unit facilities and facilitated integration within the government essential supplies delivery system, National Medical Stores in Uganda, which supports last-mile delivery to facilities. An implementing partner purchased initial raw materials necessary for production. The cost of materials for local production was compared to the price of commercial ABHR available in Uganda. **Results:** Between January and August 2021, 23 staff members were trained, and 380 batches of quality-assured ABHR (17,820 L) were produced and distributed to 278 health facilities. Consumption of ABHR in the first distribution was used to benchmark predicted ABHR consumption per targeted facility in subsequent months. Increased demand for ABHR due to the COVID-19 pandemic and the Ebola virus disease outbreak in central Uganda (September 2022) was addressed through emergency requests on a case-by-case basis. ABHR local production costs $3 per liter for materials, less than half of commercial ABHR ($8 per liter). **Conclusions:** Early results suggest that this approach is potentially sustainable but requires national advocacy as well. Leveraging existing distribution systems while building local capacity for ABHR production and distribution may improve longevity of such innovations in similar resource-limited settings.

**Disclosure:** None

